# Partial facial duplication (diprosopus): a  case report and review of the literature

**DOI:** 10.1186/s13256-024-04423-4

**Published:** 2024-03-23

**Authors:** Fathia Omer Salah, Yohannes Girma Zewdie, Semienew Ambachew, Amal Saleh Nour, Tewodros Endale

**Affiliations:** 1 Department of Radiology, Addis Ababa University, Addis Ababa, Ethiopia; 2 Division of Neonatology, Department of Pediatrics and Child Health, Addis Ababa University, Addis Ababa, Ethiopia

**Keywords:** Diprosopus, Craniofacial duplication, Corpus callosum agenesis, Interhemispheric lipoma

## Abstract

**Background:**

Diprosopus, or craniofacial duplication, is a rare entity that occurs in approximately 1 in 180,000 to 15 million live births. The degree of duplication varies from complete facial duplication to small facial structure duplication like the nose and eye. The cause of diprosopus is unknown though there are proposed factors.

**Case presentation:**

Our African patient was a term 72 hours old female neonate who was referred to our center with impression of lower facial duplication with two oral cavity that are located side to side separated by large soft tissue, she also had flat nasal bridge with widely separated nostrils and widely spaced eyes. Besides the facial malformation she had multiple episodes of vomiting with aspiration. Her blood tests were normal. Precontract brain computed tomography (CT) scan confirmed partially duplicated mandible and maxilla, two oral cavity separated by large fatty tissue, brain tissue were well formed and the only abnormality was corpus callosum agenesis and interhemispheric lipoma. In her stay at hospital nasogastric tube (NG) tube feed was initiated and started with antibiotics for aspiration pneumonia. After 25th day the neonatal passed away with possible cause of death being respiratory failure.

**Conclusion:**

Craniofacial duplication is a very rare anomaly with only a few cases reported. Most of these patients are stillborn, even if they survive the prognosis is often poor. Early prenatal diagnosis is very important as termination of pregnancy can sometimes be considered an option.

## Introduction

The “Diprosopus” (from Greek: di-two; prosopon-face) is the duplication of facial structures in a single head. Diprosopus is considered a subtype of conjoined twin. However, the pathogenesis of this anomaly is still unknown [1]. Two possible mechanisms leading to diprosopus formation have been proposed. The first mechanism is possible cranial bifurcation of the notochord during neurulation. Bifurcation causes two vertebral axes and neural plates to develop alongside each other. Another proposal is an increase in the expression of the sonic hedgehog protein, which is essential for craniofacial patterning during development [2]. Advanced maternal age, polyhydramnious, and consanguineous marriage are considered high‑risk factors for diprosopus. Prenatal diagnosis using ultrasonography, computed tomography (CT) scan, and magnetic resonance imaging (MRI) is possible. If diagnosis is made early during pregnancy, termination of pregnancy is sometimes considered an option. Usually, diprosopus patients are stillborn if not the prognosis is poor [3].

## Case report

A 72 hour-old term African new born to a 30-year-old Para—II mother presented with sign of neonatal sepsis and lower facial malformation. She had antenatal care (ANC) follow-up, but no obstetric ultrasound was performed. There was no consanguinity between her and her husband.

The neonate was 2700 g female with an APGAR score of 5 and 7 at the first and 5th minutes, respectively.

The neonate was referred to our institution for better evaluation and management of facial malformation.

**Fig. 1:**
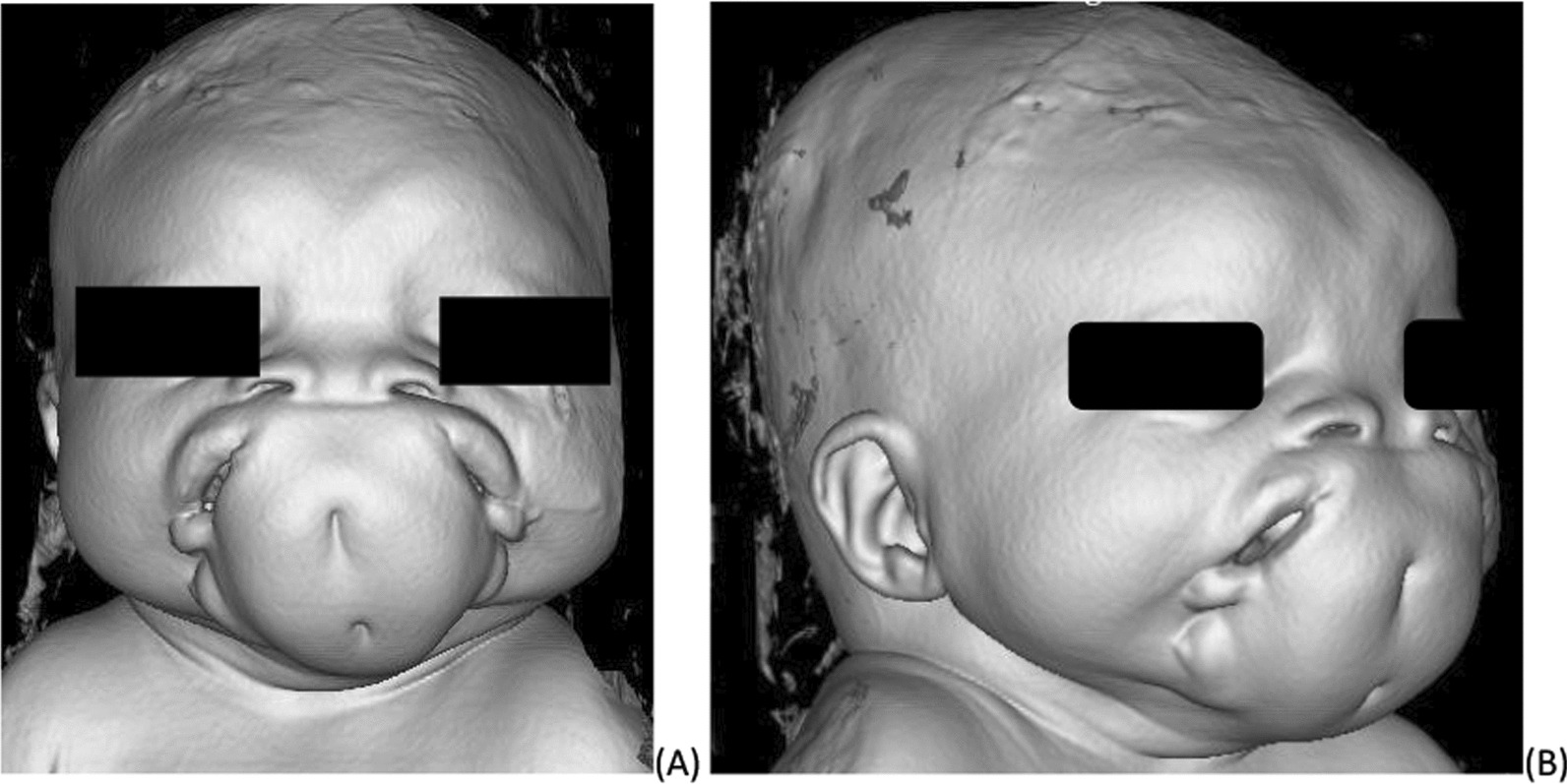
3D reconstructed image showing two oral cavity that are separated by large soft tissue having two dimples. On the same image the nostrils are seen separated by flat nasal bridge and there is increased gap between the eyes

On physical examination, her vital signs were all within the normal range, she had a depressed nasal bridge, and two nasal openings which were wide apart. There were two eyes that were also widely separated. Two mouths separated by skin covered tissue with two dimples noted and two tongues were seen (Fig. 1).

**Fig. 2:**
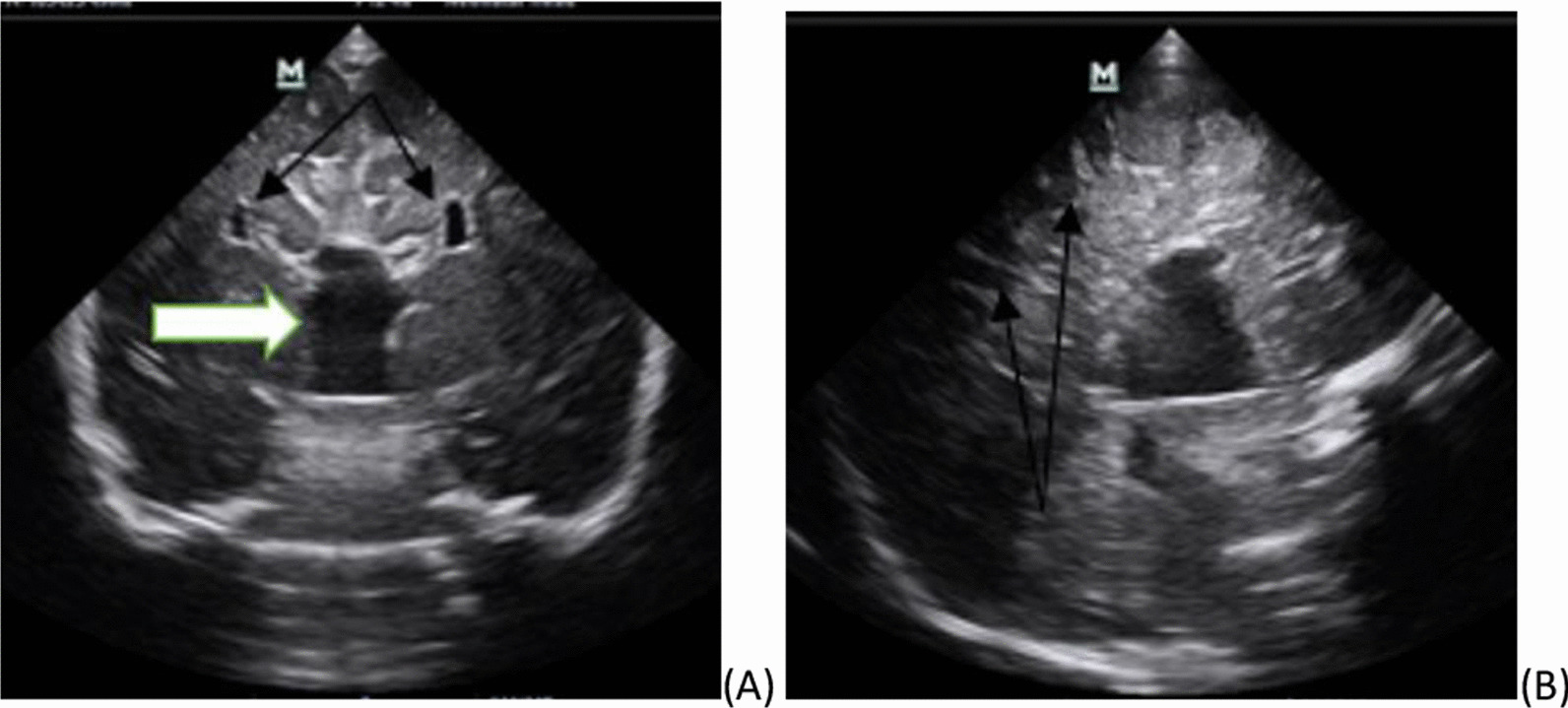
Transfontanelle ultrasound images **A** coronal image showing absence of corpus callosum with Viking helmet appearance of the anterior horn of lateral ventricles (black arrows) and dilated high riding 3rd ventricle (white arrow), **B** mid sagittal image absence of corpus callosum and radiating appearance of the grey matters typical for the sun ray appearance (arrows)

**Fig. 3:**
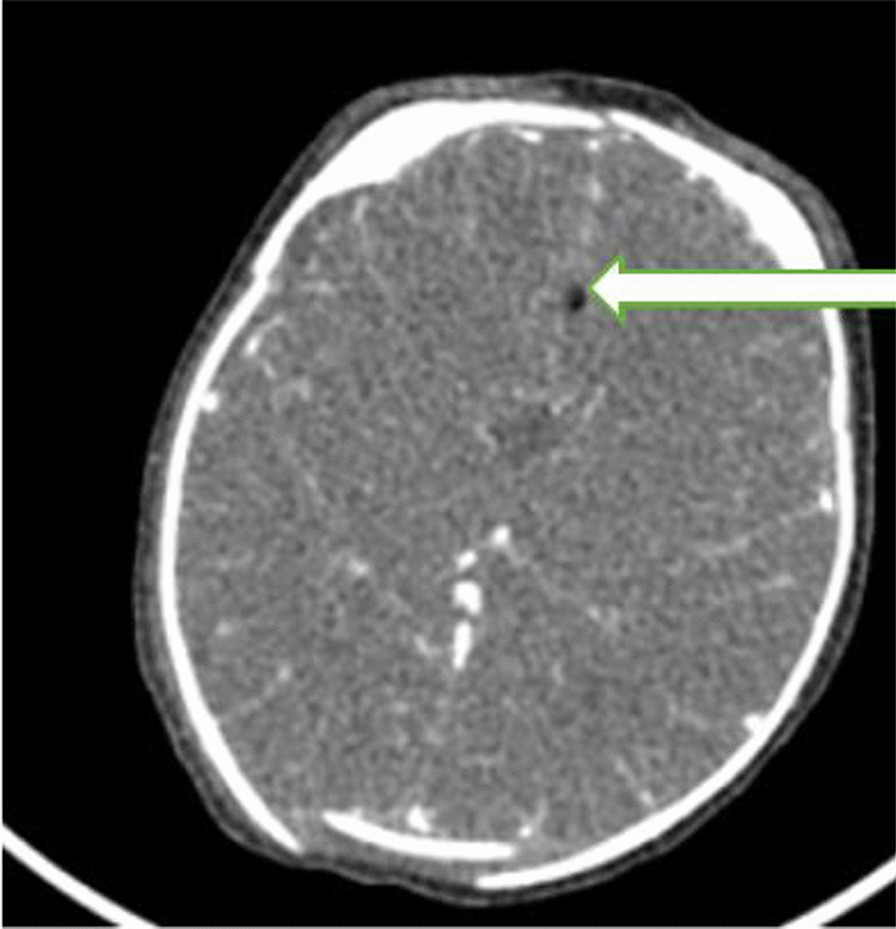
Post contrast axial brain CT image showing small midline fat density lesion representing lipoma (white arrow)

**Fig. 4:**
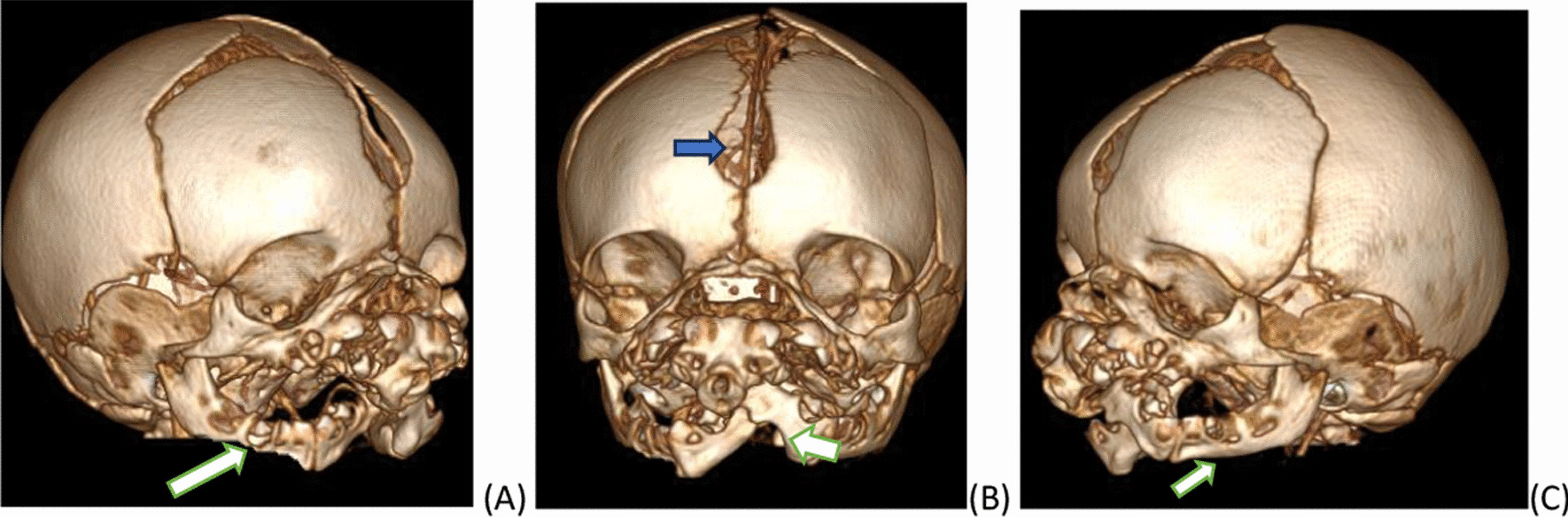
3D volume rendered bone window image of head CT [right oblique (**A**), frontal (**B**) and left oblique view (**C**)] demonstrating widely separated metopic suture (blue arrow), orbits and partially duplicated mandible (white arrow)

**Fig. 5:**
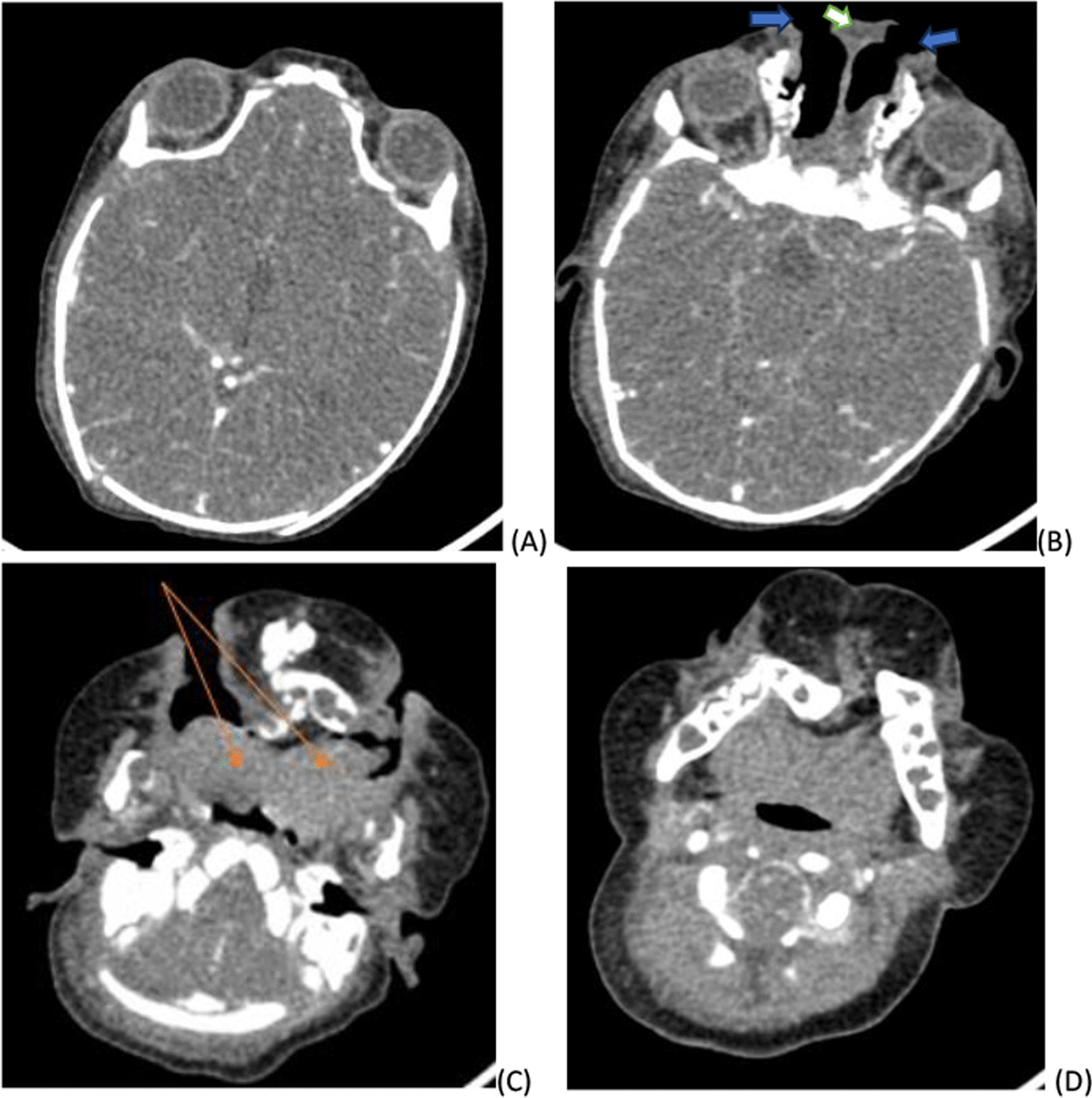
Axial soft tissue window CT image **A** showing widely separated orbits **B** more lower down images demonstrating two nasal opening (blue arrow) that are separated by soft tissue (white arrow) **C** axial image demonstrating partially duplicated tongues anteriorly (orange arrows) which fuse posteriorly directing to the two mouths on each side **D** axial image at levels of mandibles demonstrating posteriorly fussed lateral diverting tongue bases, no duplication of the posterior aero digestive system is noted

Blood test results and imaging were also recorded. All the blood test results were normal. Pertinent imaging findings with transfontanelle ultrasound, head and neck CT. Transfontanelle ultrasound demonstrate normal well-formed brain tissue with normal ventricular size, the only positive finding was non visualization of the corpus callosum (Fig. 2). Head and neck CT findings were widely separated orbits and nostrils. There was also small interhemispheric fat attenuating lesion, which suggested lipoma (Fig. 3)  Metopic suture was widened, partial duplicated maxilla and mandible covered by redundant subcutaneous tissue was noted  (Fig. 4). There was also duplication of the anterior two-thirds of the tongue (Fig. 5).

While the neonate was on treatment for early onset neonatal sepsis with antibiotics and nasogastric tube feeding, she developed multiple episodes of vomiting and aspiration complicated with aspiration pneumonia. Despite the treatment given, the neonate passed away on the 25th day after admission possibly because of respiratory failure secondary to aspiration pneumonia.

## Discussion

Diprosopus is a rare clinical entity with very few reported cases in the literature. There are only approximately 36 reported cases in the literature [3].

There is a predominance of females over males (2:1) [4]. The duplication can involve structures as small as the nasal to complete facial structures [5]. A complete duplication or dicephalus is associated with a high incidence of anomalies in the central nervous system (CNS), cardiovascular system (CVS), gastrointestinal system (GI) and respiratory system (RS), as well as in the cleft lip and palate. Partial duplication is less often associated with other anomalies. Infants with partial duplication have a mandible and a mouth, which are most duplicated. The CNS anomalies involve anencephaly, duplication of the brain with two prosencephalon and a single rhombencephalon, two diencephalons (each with a set of thalami and basal ganglia) and two symmetric telencephalons (each with a set of cerebral hemispheres and lobes). Hypoplasia of the medial temporal lobe was also noted. Multiple spinal abnormalities with duplication of the cervical spine and abnormal cervical and thoracic vertebrae have been observed [6]. The defects in the other organs include diaphragmatic hernia; cardiac defects (VSD, an overriding aorta and a hypoplastic ascending and descending aorta; an aortic arch; and dextrocardia); bilateral dysplastic cystic kidneys; hypoplasia of the ureters and the urinary bladder cleft lip palate and imperforate anus [7, 8].

The embryology of this condition has been a matter of debate. The most accepted theory is that conjoined twins result from an embryological disturbance in the separation of the twins during the 2nd week of pregnancy (12–13 days) as a result of the abnormal splitting of post-implantation blastocytes [9]. Such incomplete, separated, germinal discs lead to this extremely rare fetal anomaly. However, recently, it has been postulated that conjoined twins result from the development of two independent notochords, which were initially destined to become separate twins but were too close to develop independently [10].

The earliest clinical finding associated with diprosopus is polyhydramnious [11]. The disease can be diagnosed prenatally by ultrasonography, CT scan, and MRI which reveal all the facial features and associated anomalies but these facilities are not widely available in developing countries. Estimation of serum alpha fetoprotein levels also helps in prenatal diagnosis.

The prognosis depends on the degree of duplication, as cases with complete duplication are still born and those with partial duplication vary from early neonatal death from primary or associated anomalies or acquired disease to possible long term survival after surgical correction [6, 12]. With the spread of prenatal follow-up, early detection of cases with conjoined twins, such as diprosopus, is essential for social, economic and ethical reasons and will enable parents to make decisions in the early weeks [13].

## Conclusions

Craniofacial duplication is a rare entity that is more common in females. Pathophysiology is incompletely understood but the most accepted theory is that conjoined twins result from an embryological disturbance in the separation of the twins during the 2nd week of pregnancy. In partial facial duplication CT and MR imaging are important for evaluating the degree of duplication so that cosmetic reconstruction can be planned.

## Data Availability

Not applicable.
